# Respiratory control during air-breathing exercise in humans following an 8 h exposure to hypoxia

**DOI:** 10.1016/j.resp.2008.06.008

**Published:** 2008-08-31

**Authors:** Mari Herigstad, Marzieh Fatemian, Peter A. Robbins

**Affiliations:** Department of Physiology, Anatomy and Genetics, Parks Road, University of Oxford, Oxford OX1 3PT, United Kingdom

**Keywords:** Exercise, Hypoxia, Ventilation

## Abstract

Hypoxic exposure lasting a few hours results in an elevation of ventilation and a lowering of end-tidal $P_{\text{C}{\text{O}}_{2}}(P_{\text{E}{\text{T}}_{\text{C}{\text{O}}_{2}}})$ that persists on return to breathing air. We sought to determine whether this increment in ventilation is fixed (hypothesis 1), or whether it increases in proportion to the rise in metabolic rate associated with exercise (hypothesis 2). Ten subjects were studied on two separate days. On 1 day, subjects were exposed to 8 h of isocapnic hypoxia (end-tidal $P_{{\text{O}}_{2}}$ 55 Torr) and on the other day to 8 h of euoxia as a control. Before and 30 min after each exposure, subjects undertook an incremental exercise test. The best fit of a model for the variation in $P_{\text{E}{\text{T}}_{\text{C}{\text{O}}_{2}}}$ with metabolic rate gave a residual squared error that was ∼20-fold less for hypothesis 2 than for hypothesis 1 (*p* < 0.005, *F*-ratio test). We conclude that the alterations in respiratory control induced during early ventilatory acclimatization to hypoxia better reflect those associated with hypothesis 2 rather than hypothesis 1.

## 1 Introduction

Tansley et al. (1997) demonstrated that after an 8-h exposure to hypoxia, there was an increase in ventilation $\left({\dot{V}}_{\text{E}}\right)$ that persisted following a subsequent 8-h period of breathing air. This finding was the same whether or not the respiratory alkalosis that normally accompanies an exposure to hypoxia was prevented by adding CO_2_ to the inspirate to maintain eucapnia over the 8-h period.

Of themselves, these findings do not indicate whether the 8-h exposure to hypoxia generates a fixed increase in ${\dot{V}}_{\text{E}}$ irrespective of metabolic rate (hypothesis 1), or whether the exposure generates a fixed increase in the ratio of ventilation to metabolic rate (hypothesis 2). If hypothesis 1 is correct, then this would imply that the mechanisms underlying the initial process of ventilatory acclimatization to hypoxia and those underlying the respiratory response to exercise are independent and additive in their effects on ventilation. Alternatively, if hypothesis 1 is not correct, then these two mechanisms affecting ventilation must interact to some degree with each other. The simplest form of such interaction would be a straightforward multiplicative interaction and this is used as our competing hypothesis (hypothesis 2).

In order to distinguish these hypotheses, we compared the responses to incremental exercise before and after an 8-h exposure to isocapnic hypoxia. If there is an increase in ${\dot{V}}_{\text{E}}$ which is fixed irrespective of metabolic rate (hypothesis 1), then we predict that the difference in end-tidal $P_{\text{C}{\text{O}}_{2}}(P_{\text{E}{\text{T}}_{\text{C}{\text{O}}_{2}}})$ before versus after the hypoxic exposure should diminish with increasing metabolic rate, whereas if the exposure generates a fixed increase in the ratio of ventilation to metabolic rate (hypothesis 2), then we predict that this difference in $P_{\text{E}{\text{T}}_{\text{C}{\text{O}}_{2}}}$ should remain constant irrespective of metabolic rate. These two predictions are derived mathematically in Section 2 together with a suitable transform of metabolic rate that enables us best to compare the two hypotheses.

In Section 4, the results of the present study are compared with those of earlier studies examining this issue, but following longer periods of hypoxic exposure during which some degree of respiratory alkalosis – and possibly renal compensation – would also have been present.

## 2 Methods

### 2.1 Subjects

Ten healthy subjects (four women, six men, age 25 ± 3 year, mean ± S.D.) consented to take part in this study. All were non-smoking and none had a history of cardiovascular or respiratory disease. The study was approved by the Oxfordshire Clinical Research Ethics Committee and followed the guidelines set in the Declaration of Helsinki.

### 2.2 Protocols

Each subject undertook two protocols. The protocols were undertaken on separate days and the order in which each subject undertook the two protocols was random. There was always an interval of at least 1 week between protocols to ensure that the protocols had no effect on each other. Protocols always started at the same time of day. To reduce variability, female subjects were either always studied 1 week after the onset of menstruation or, if using a monophasic oral contraceptive, during a period when they were taking the active component.

The subject's resting $P_{\text{E}{\text{T}}_{\text{C}{\text{O}}_{2}}}$ was measured before the start of each experiment. In the hypoxia protocol, the subject was exposed to isocapnic hypoxia for 8 h ($P_{\text{E}{\text{T}}_{{\text{O}}_{2}}}=55\;\text{Torr},P_{\text{E}{\text{T}}_{\text{C}{\text{O}}_{2}}}$ held at the subject's normal air-breathing value) in a custom-built chamber. This level of hypoxia is known to be well tolerated by healthy subjects and also reliably to induce acclimatization over a period of 8 h (Howard and Robbins, 1995). In the control protocol, the subject breathed air for 8 h in the chamber. Subjects were not told which experiment they were undertaking on any particular day, but all subjects were able to distinguish between the air-breathing protocol and the hypoxic protocol. Subjects were not informed of the end-point measurements or the expected outcome of the study.

Before and after the 8-h period in the chamber, the subject undertook an incremental exercise test to exhaustion during which measurements of respiratory function were made. All subjects rested quietly breathing room air for 30 min after leaving the chamber prior to undertaking the second exercise test. This was to allow the subject time to recover from all rapidly reversible effects of the exposure to hypoxia whilst retaining the acclimatization response (Tansley et al., 1997).

### 2.3 Apparatus and techniques

To determine the subject's air-breathing values for $P_{\text{E}{\text{T}}_{\text{C}{\text{O}}_{2}}}$, respired gas was sampled via a catheter taped just below the subject's nostril and analysed by mass spectrometry (Airspec 2200). Respired gas obtained via the nostril has previously been shown to give values for $P_{\text{E}{\text{T}}_{\text{C}{\text{O}}_{2}}}$ that closely reflect those from respired gas sampled at the mouth (Fatemian et al., 2003).

Subjects undertook the incremental exercise seated upright on a electrically braked cycle ergometer (Mijnhardt KEM3). During exercise, subjects were asked to keep the pedalling frequency constant at 60 min^−1^ using a coloured light display mounted on the handlebars which indicated pedal rate. The protocol for the incremental exercise test was as follows: subjects pedaled at 0 W for 1 min, after which the load was increased by 15 W every minute until exhaustion. Exhaustion was considered as the point when either the subject stopped voluntarily or was unable to maintain a pedal rate of 60 rpm, and was told to stop.

During the incremental exercise test, the subject breathed through a mouthpiece assembly with their nose occluded. The respired gas was sampled via a fine catheter from a port in the mouthpiece close to the subject's mouth and analysed continuously by mass spectrometry. Respiratory volumes were measured by means of a turbine volume-measuring device incorporated in the mouthpiece assembly. Respiratory flows and timing information were recorded using a pneumotachograph. The gas signals and the inspiratory and expiratory volume signals were passed to a data acquisition computer. This apparatus has been described in detail elsewhere (Robbins et al., 1982).

Both the hypoxia protocol and the control protocol were undertaken in a custom-built chamber large enough to allow the subject to move around comfortably during the experiment. The subject wore a nasal catheter at all times whilst in the chamber, through which respired gases were continuously sampled and analyzed for $P_{\text{C}{\text{O}}_{2}}$ and $P_{{\text{O}}_{2}}$. The subjects also wore a pulse oximeter which continuously monitored the arterial oxygen saturation. The composition of the gas within the chamber was automatically adjusted every 5 min by a computer, or at manually overridden intervals. Both $P_{\text{E}{\text{T}}_{\text{C}{\text{O}}_{2}}}$ and $P_{\text{E}{\text{T}}_{{\text{O}}_{2}}}$ were regulated in this manner. The system has been described in detail elsewhere (Howard et al., 1995). Subjects were under constant observation by the experimenter during their time in the chamber.

### 2.4 Data analysis

The breath-by-breath data for the incremental exercise tests were averaged for each minute of exercise. The values were interpolated at percentages of maximal work rate, starting at 0% of maximal work rate with increments of 5%. This generated an equal number of data points for each volunteer, 21 data points in total. The effect of the 8-h chamber exposures (hypoxia and control) on the responses in $P_{\text{E}{\text{T}}_{\text{C}{\text{O}}_{2}}}$ and ${\dot{V}}_{\text{E}}$ to incremental exercise was assessed using repeated-measures analysis of variance (ANOVA). In the theory section below, two alternative relationships between the magnitude of the effect of acclimatization on alveolar $P_{\text{C}{\text{O}}_{2}}$ and metabolic rate are derived for the two competing hypotheses of the study. For each hypothesis, the best-fit parameter for the model was determined through linear regression and the residual squared error used as an index of goodness of fit. Statistical significance was assumed at *p* < 0.05 for this study. The statistical package SPSS was used for the analysis.

### 2.5 Theory—alternative hypotheses

The alternative hypotheses of this paper are: (i) that an 8-h exposure to hypoxia induces a fixed increment in ventilation, and (ii) that an 8-h exposure to hypoxia induces an increment in ventilation that is proportional to metabolism. This section calculates the expected effect of each hypothesis on the variation in $P_{\text{E}{\text{T}}_{\text{C}{\text{O}}_{2}}}$ with metabolic rate. Symbols are: ${\dot{V}}_{\text{A}}$, alveolar ventilation before acclimatization; ${{\dot{V}}^{\prime }}_{\text{A}}$, alveolar ventilation after acclimatization; ${\dot{V}}_{\text{C}{\text{O}}_{2}}$, CO_2_ production; $P_{\text{AC}{\text{O}}_{2}}$, alveolar $P_{\text{C}{\text{O}}_{2}}$ before acclimatization; ${P^{\prime }}_{\text{AC}{\text{O}}_{2}}$, alveolar $P_{\text{C}{\text{O}}_{2}}$ after acclimatization; and PB barometric pressure.

#### 2.5.1 Hypothesis 1—fixed increment in ventilation

Let the increment in ${\dot{V}}_{\text{A}}$ following acclimatization be h (i.e., ${{\dot{V}}^{\prime }}_{\text{A}}={\dot{V}}_{\text{A}}+h$). Using mass balance, before acclimatization, we may write:

(1) $${\dot{V}}_{\text{C}{\text{O}}_{2}}=\frac{{\dot{V}}_{\text{A}}P_{\text{AC}{\text{O}}_{2}}}{P_{\text{B}}}$$

Following acclimatization, we may write:

(2) $${\dot{V}}_{\text{C}{\text{O}}_{2}}=\frac{({\dot{V}}_{\text{A}}+h){P^{\prime }}_{\text{AC}{\text{O}}_{2}}}{{\text{P}}_{\text{B}}}$$

Combining equations, eliminating ${\dot{V}}_{\text{A}}$ and tidying yields:

(3) $$\frac{{P^{\prime }}_{\text{AC}{\text{O}}_{2}}-P_{\text{AC}{\text{O}}_{2}}}{P_{\text{AC}{\text{O}}_{2}}}=\frac{-h{P^{\prime }}_{\text{AC}{\text{O}}_{2}}}{P_{\text{B}}{\dot{V}}_{\text{C}{\text{O}}_{2}}}$$

#### 2.5.2 Hypothesis 2—increment in ventilation proportional to metabolic rate

Let the proportionate increment in ${\dot{V}}_{\text{A}}$ following acclimatization be *k* (i.e., ${{\dot{V}}^{\prime }}_{\text{A}}=k{\dot{V}}_{\text{A}}$). Using mass balance, before acclimatization, we may write:

(4) $${\dot{V}}_{\text{C}{\text{O}}_{2}}=\frac{{\dot{V}}_{\text{A}}P_{\text{AC}{\text{O}}_{2}}}{P_{\text{B}}}$$

Following acclimatization, we may write:

(5) $${\dot{V}}_{\text{C}{\text{O}}_{2}}=\frac{k{\dot{V}}_{\text{A}}{P^{\prime }}_{\text{AC}{\text{O}}_{2}}}{P_{\text{B}}}$$

Combining equations, eliminating ${\dot{V}}_{A}$ and tidying yields:

(6) $$\frac{{P^{\prime }}_{\text{AC}{\text{O}}_{2}}-P_{\text{AC}{\text{O}}_{2}}}{P_{\text{AC}{\text{O}}_{2}}}=-\frac{k-1}{k}$$

#### 2.5.3 Comparison of hypotheses

In Eq. (3), the term *h* may be estimated by regression for values of $P_{\text{AC}{\text{O}}_{2}}$ and ${P^{\prime }}_{\text{AC}{\text{O}}_{2}}$ at different values of ${\dot{V}}_{\text{C}{\text{O}}_{2}}$. In Eq. (6), the term *k* may be estimated by simple averaging of the values obtained at different values of ${\dot{V}}_{\text{C}{\text{O}}_{2}}$ (since these are not dependent upon ${\dot{V}}_{\text{C}{\text{O}}_{2}}$). The hypotheses may then be compared through the squared error between the model predictions and actual values for $({P^{\prime }}_{\text{AC}{\text{O}}_{2}}-P_{\text{AC}{\text{O}}_{2}})\text{/}P_{\text{AC}{\text{O}}_{2}}$.

In the experimental work, values have been obtained for $P_{\text{E}{\text{T}}_{\text{C}{\text{O}}_{2}}}$ rather than $P_{\text{AC}{\text{O}}_{2}}$ and the relationship between these is known to vary systematically with exercise. However, as we are principally interested in the quantity ${P^{\prime }}_{\text{AC}{\text{O}}_{2}}-P_{\text{AC}{\text{O}}_{2}}$, the effects of any differences between $P_{\text{E}{\text{T}}_{\text{C}{\text{O}}_{2}}}$ and $P_{\text{AC}{\text{O}}_{2}}$ will tend to cancel.

## 3 Results

Of the 10 subjects originally recruited to the study, all completed both the control protocol and the main protocols successfully and provided data that were suitable for analysis. No subject undertook the second protocol within a week of the first, and no subject took longer than 2 months to complete both protocols. Table 1 lists the physical characteristics of each subject, and includes the maximum work rate attained by the subject during incremental exercise together with their maximal oxygen uptake capacity $\left({\dot{V}}_{{\text{O}}_{2}\text{max}}\right)$. Neither maximal work rate nor ${\dot{V}}_{{\text{O}}_{2}}$ differed significantly before versus after the 8-h chamber exposures for either the air-breathing or hypoxia protocols. Values for the change in $P_{\text{E}{\text{T}}_{\text{C}{\text{O}}_{2}}}$ (p.m.–a.m.) measured under resting conditions following exposure to 8 h of sustained hypoxia are also included in the table. Out of the 10 subjects, only 1 did not show a reduction in $P_{\text{E}{\text{T}}_{\text{C}{\text{O}}_{2}}}$ after the hypoxia protocol, whereas with the control protocol, 7 subjects showed an increase in $P_{\text{E}{\text{T}}_{\text{C}{\text{O}}_{2}}}$ and 3 subjects a reduction. The average fall in resting air-breathing $P_{\text{E}{\text{T}}_{\text{C}{\text{O}}_{2}}}$ after the 8-h chamber exposure to hypoxia was 2.7 ± 0.5 Torr (mean ± S.E., *p* < 0.01), whereas the average fall in resting $P_{\text{E}{\text{T}}_{\text{C}{\text{O}}_{2}}}$ after the 8-h chamber exposure to air was −0.1 ± 0.4 Torr (mean ± S.E., NS).

**Fig. 1:**
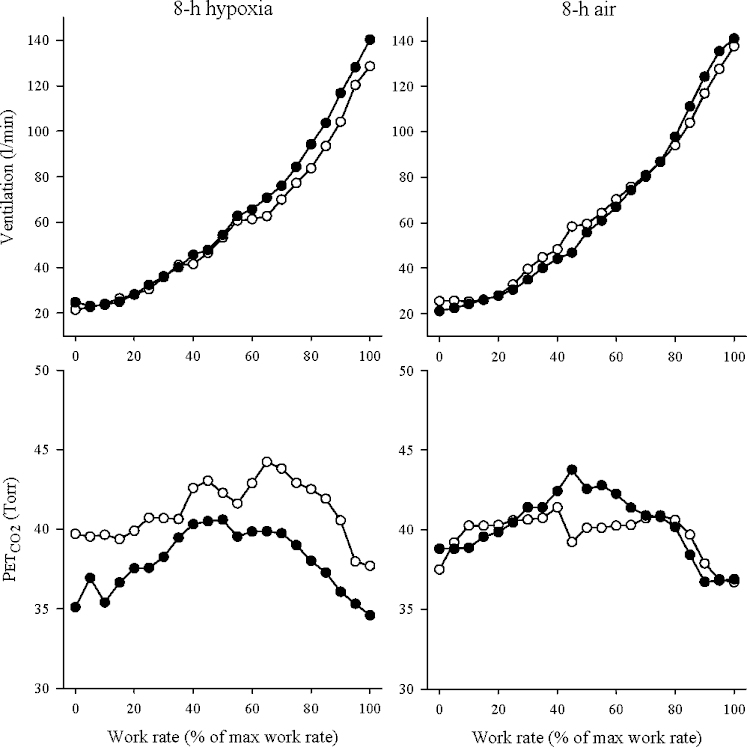
Ventilation and end-tidal $P_{\text{C}{\text{O}}_{2}}(P_{\text{E}{\text{T}}_{\text{C}{\text{O}}_{2}}})$ during incremental exercise before and after 8-h chamber exposure for one subject (1). Left, 8-h chamber exposure to isocapnic hypoxia; right, 8-h chamber exposure to air. Open symbols, incremental exercise test before 8-h exposure (a.m. values); closed symbols, incremental exercise test after 8-h exposure (p.m. values). In the subject illustrated, maximal work rate was 285 W.

Fig. 1 illustrates, for one subject, the respiratory responses to the incremental exercise tests before and after the 8-h chamber exposures to hypoxia and to air. A similar pattern of response for ${\dot{V}}_{\text{E}}$ was observed in all tests. Despite this, following the 8-h exposure to hypoxia, the reduction in $P_{\text{E}{\text{T}}_{\text{C}{\text{O}}_{2}}}$ observed at rest persisted throughout the incremental exercise test. By comparison, the 8-h chamber exposure to air did not generate any consistent changes in $P_{\text{E}{\text{T}}_{\text{C}{\text{O}}_{2}}}$.

**Fig. 2:**
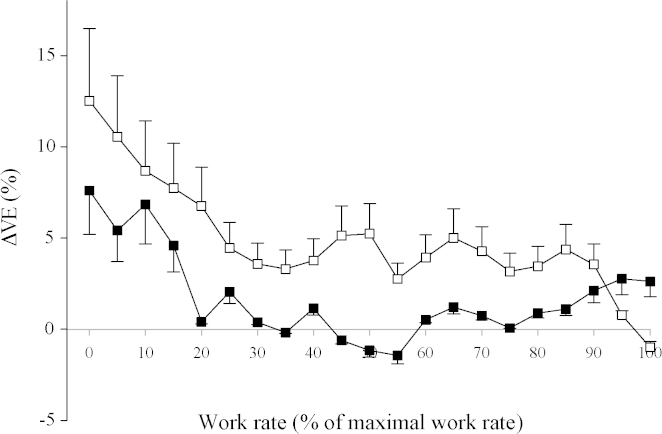
The percentage change in ventilation $\left(\Delta {\dot{V}}_{\text{E}}\right)$ following an 8-h chamber exposure as a function of work rate measured during incremental exercise. $\Delta {\dot{V}}_{\text{E}}$ was calculated as the percentage change in ${\dot{V}}_{\text{E}}$ after versus before the 8-h chamber exposures at each work rate. Values are averages across all subjects and error bars indicate S.E. Open symbols, hypoxia protocol; closed symbols, control protocol.

Fig. 2 illustrates the average increment in ${\dot{V}}_{\text{E}}$ following the 8-h chamber exposures across all subjects, where the increment is the percentage increase in ${\dot{V}}_{\text{E}}$ following the exposure compared with the pre-exposure value. ${\dot{V}}_{\text{E}}$ appears ∼5% higher following an 8-h exposure to hypoxia and this difference was maintained throughout incremental exercise, at least to 90% of the maximum exercise capacity. This was not the case following the 8-h control protocol. However, repeated measures ANOVA did not demonstrate that the difference between protocols was significant.

**Fig. 3:**
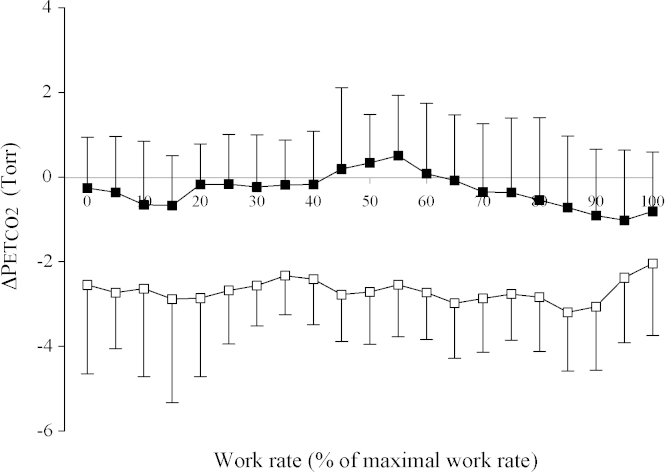
The change in end-tidal $P_{\text{C}{\text{O}}_{2}}(\Delta P_{\text{E}{\text{T}}_{\text{C}{\text{O}}_{2}}})$ following an 8-h chamber exposure as a function of work rate measured during incremental exercise. Values are averages across subjects and error bars indicate S.E. $\Delta P_{\text{E}{\text{T}}_{\text{C}{\text{O}}_{2}}}$ was calculated as the difference between $P_{\text{E}{\text{T}}_{\text{C}{\text{O}}_{2}}}$ values measured after and before the 8-h chamber exposures (p.m.–a.m.). Open symbols, hypoxia protocol; closed symbols, control protocol.

Fig. 3 illustrates the average difference in $P_{\text{E}{\text{T}}_{\text{C}{\text{O}}_{2}}}$ after the 8-h chamber exposures compared with before the exposures. $P_{\text{E}{\text{T}}_{\text{C}{\text{O}}_{2}}}$ appears ∼2 Torr lower following the 8-h exposure to hypoxia compared with before the exposure, and this difference was maintained throughout the incremental exercise test. No such differences were detectable for the control protocol. Repeated measures ANOVA demonstrated that this difference between the protocols was significant (*p* < 0.01).

**Fig. 4:**
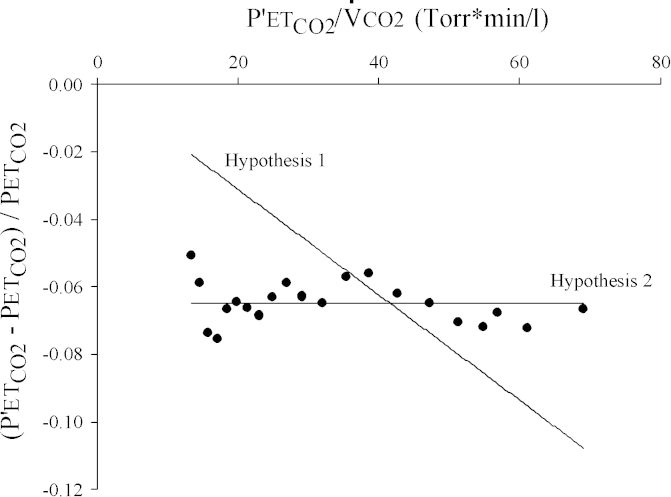
Fractional change in $P_{\text{E}{\text{T}}_{\text{C}{\text{O}}_{2}}}$ plotted against a transform of CO_2_ production $\left({\dot{V}}_{\text{C}{\text{O}}_{2}}\right)$. Experimental data are averages across volunteers and shown by the closed symbols. The straight line labelled hypothesis 1 reflects the best fit of a model to the data that assumes the increment in ventilation induced following 8-h of hypoxia is fixed. The straight line labelled hypothesis 2 reflects the best fit of a model to the data that assumes the increment in ventilation induced following 8-h of hypoxia is proportional to ${\dot{V}}_{\text{C}{\text{O}}_{2}}$. The mean squared error associated with hypothesis 1 is 0.000811 and hypothesis 2 is 0.000039 (*p* < 0.005 for difference, *F*-ratio test with degrees of freedom corrected for between correlation residuals (Chipman et al., 1968; Kramer, 1980)). $P_{\text{E}{\text{T}}_{\text{C}{\text{O}}_{2}}}$ reflects the end-tidal $P_{\text{C}{\text{O}}_{2}}$ before acclimatization and ${P^{\prime }}_{\text{E}{\text{T}}_{\text{C}{\text{O}}_{2}}}$ reflects the end-tidal $P_{\text{C}{\text{O}}_{2}}$ after acclimatization.

Fig. 4 illustrates the fractional change in $P_{\text{E}{\text{T}}_{\text{C}{\text{O}}_{2}}}$ following acclimatization plotted against a transform of the CO_2_ production. In this plot, a change in alveolar ventilation post-acclimatization that is fixed irrespective of metabolic rate would produce a linear relationship between the two variables that is constrained to pass through the origin. In contrast, a change in alveolar ventilation post acclimatization that is proportional to metabolic rate would produce a change that is independent of the transform of CO_2_ production (i.e., the relationship is constrained to be a line of zero slope). Fig. 4 illustrates the best fit of each of these two relationships to the data. In the figure, the second model, where the post-acclimatization increase in alveolar ventilation is proportional to metabolism, clearly fits the data better. This is confirmed by a residual mean squared error that is lower by a factor of ∼20 compared with the model where the post-acclimatization increase in alveolar ventilation is fixed, regardless of metabolic rate. This difference in mean squared error is significant at *p* < 0.005 using an F-ratio test after degrees of freedom had been corrected for the presence of auto correlation within the residuals (Chipman et al., 1968; Kramer, 1980).

## 4 Discussion

The reduction in air-breathing $P_{\text{E}{\text{T}}_{\text{C}{\text{O}}_{2}}}$ at rest following an 8 h exposure to hypoxia was broadly consistent with that observed in previous studies using such an exposure to hypoxia (Fatemian and Robbins, 1998, 2001). The major finding from this study was that the reduction in $P_{\text{E}{\text{T}}_{\text{C}{\text{O}}_{2}}}$ remained effectively unaltered during incremental exercise to exhaustion. This relatively constant reduction in $P_{\text{E}{\text{T}}_{\text{C}{\text{O}}_{2}}}$ implies that early VAH does not involve a fixed increase in ${\dot{V}}_{\text{E}}$, but rather generates an increment in ${\dot{V}}_{\text{E}}$ that is in proportion to metabolism.

### 4.1 Comparison with other studies examining the effect of VAH on air-breathing exercise

A number of investigators have examined the effect on the ventilatory responses to air-breathing exercise of longer exposures to hypoxia than those employed in the present study. Dempsey et al. (1972) measured air-breathing ${\dot{V}}_{\text{E}}$ during exercise both at sea-level and after 45 days of VAH (3100 m). They observed an increase in resting ${\dot{V}}_{\text{E}}$ of ∼1.5 L/min under air-breathing conditions following 45 days of VAH, and this increase was further enhanced during mild to moderate air-breathing exercise (but not maximal exercise). Forster and Klausen (1973) studied the response to air-breathing exercise following a 5–7-day period of either metabolic acidosis or VAH. A lowered arterial $P_{\text{C}{\text{O}}_{2}}$ was observed after both metabolic acidosis and VAH, and this lower arterial $P_{\text{C}{\text{O}}_{2}}$ was defended when the subjects were re-exposed to air-breathing exercise. Bebout et al. (1989) measured air-breathing ${\dot{V}}_{\text{E}}$ at sea level both at rest and during various levels of exercise before and after a 2-week period of VAH (3800 m). The reduction in resting arterial $P_{\text{C}{\text{O}}_{2}}$ of ∼6 Torr following acclimatization was almost unaltered by undertaking exercise at 30, 60 and 90% of the work rate associated with maximum O_2_ uptake capacity. Green et al. (2000) examined the respiratory response to exercise in 5 healthy subjects before and after a 21 day expedition to altitude (6194 m). They did not make any observations in relation to $P_{\text{E}{\text{T}}_{\text{C}{\text{O}}_{2}}}$, but did observe that ${\dot{V}}_{\text{E}}$ during incremental exercise after VAH was significantly higher compared with before the expedition. It is not clear from their results whether the increment in ${\dot{V}}_{\text{E}}$ following VAH was constant at all work rates, or whether it increased in proportion to work rate.

Despite the apparent similarities between these earlier findings and those of the present study, there are important differences relating to the nature and duration of the hypoxic exposures. In each of the previous studies, the alveolar $P_{\text{C}{\text{O}}_{2}}$ was allowed to fall naturally during the course of the exposure, and the duration of the exposure would have been sufficient to allow substantial systemic alterations in metabolic acid base status to occur (Kellogg, 1963; Severinghaus et al., 1963; Forster et al., 1975). In contrast, the hypoxic exposures in the present study were both isocapnic and of too short a duration to induce such changes in acid base status (Cruz et al., 1980; Tansley et al., 1998). Thus, the present study indicates that neither a significant alteration in acid base balance nor a persistent alteration in resting ${\dot{V}}_{\text{E}}$ of several days duration are required in order for the ventilatory response to exercise to change in response to alterations in resting ${\dot{V}}_{\text{E}}\text{/alveolar}\;P_{\text{C}{\text{O}}_{2}}$.

Other investigators have considered whether prior sustained exposure to intermittent hypoxia would produce similar effects on the ventilatory response to exercise under air-breathing conditions, but these studies have yielded varying results. Katayama et al. (2002, 2004) and Foster et al. (2006) found that prior exposure to various forms of intermittent hypoxia (1 h per day for 7 days; 3 h per day for 14 days; and 30 min per day for 12 days either as one exposure or as 12 sets of 5-min exposures) had no significant effect on the ventilatory response to incremental exercise to exhaustion. On the other hand, Gore et al. (2001) and Townsend et al. (2005) exposed subjects to nocturnal hypoxia (9.5 h per night for 23 nights, and 8–10 h per night for 20 nights) and observed a subsequent increase in ${\dot{V}}_{\text{E}}$ during dynamic exercise.

### 4.2 Peripheral chemoreflex and early acclimatization to hypoxia

There is now a substantial body of evidence to suggest that the early stages of VAH may arise through a progressive alteration in peripheral chemoreflex function (reviewed by Robbins, 2007). If correct, then this would suggest that the effects of acclimatization on the ventilatory response to exercise may have some broad similarity to the effects of peripheral chemoreceptor stimulation on the ventilatory response to exercise.

In the case of acute hypoxia as a peripheral chemoreceptor stimulant, there is a well-recognised interaction between its efficacy as a stimulus to ventilation and the degree of muscular exercise being undertaken—a result consistent with the present study. Indeed, Wood et al. (2007) have demonstrated that this interaction is itself affected (reduced) by a period of prior VAH, and conclude that there may be some interrelation between these processes.

Other studies have looked at ${\dot{V}}_{\text{E}}$ during exercise after manipulating the peripheral chemoreflex sensitivity pharmacologically rather than exposing the subjects to hypoxia. Almitrine and domperidone are substances that augment peripheral chemoreflex sensitivity. Dopamine, on the other hand, attenuates peripheral chemoreflex sensitivity.

Schaefer and Mitchell (1989) examined the effects of an intravenous infusion of domperidone (0.5 mg/kg) on respiratory control in goats under conditions of both rest and exercise. They found that almitrine induced a ∼5 Torr reduction in arterial $P_{\text{C}{\text{O}}_{2}}$ at rest, and that this fell by a further ∼1.5 Torr on exercise, in keeping with a goat's mildly hypocapnic response to undertaking exercise. In humans, Naeije et al. (1993) demonstrated that oral intake of almitrine (75 mg) resulted in a small but significant reduction in $P_{\text{AC}{\text{O}}_{2}}$ during exercise.

By way of contrast, intravenous infusions of dopamine (3 μg kg^−1^ min^−1^) in humans, which should lower peripheral chemoreflex sensitivity, did not significantly alter ${\dot{V}}_{\text{E}}$ during air-breathing exercise, either during steady-state moderate workloads (Boetger and Ward, 1986) or during incremental exercise to exhaustion (Henson et al., 1992).

### 4.3 Implications for respiratory control

A major function of the peripheral chemoreflex is to provide feedback protection against inadequate, or indeed excessive, ventilation. For a feedback system to be effective, the closed loop gain of the system needs to be appropriate: too low, and poor control follows; too high, and instability ensues. A complication of the respiratory control system is that, as metabolic rate rises during exercise, so, in the absence of any adjustments, closed loop gain will decline and feedback will become less effective. The theory underlying this has been developed by Khoo et al. (1982), and the reduction in loop gain with O_2_ consumption/CO_2_ production is shown in Eqs. (14), (16) and (18) of their study together with the result in their Table 3. This arises as a consequence of the increase in the area constant of the metabolic hyperbola. Against this background, the increase in sensitivity of the peripheral chemoreflex with increasing levels of exercise may be seen as a compensatory mechanism to maintain the closed loop gain at a value that provides adequate feedback control. The interaction between VAH and metabolic rate in the present study has a similar characteristic in the sense that, as metabolic rate rises, the influence of VAH increases so that alveolar $P_{\text{C}{\text{O}}_{2}}$ is maintained constant.

### 4.4 Summary

The main finding of this study was that the absolute effect on respiration of a prior 8-h exposure to hypoxia varied with metabolic rate as this increased with exercise. This finding implies that the mechanisms underlying the initial process of ventilatory acclimatization to hypoxia interact to some degree with the mechanisms by which ventilation is increased during muscular exercise.

## Grants

This study was supported by the Wellcome Trust. M. Herigstad has been supported by the Norwegian State Educational Loan Fund and through a Florey EPA grant from the Queen's College, Oxford.

## Figures and Tables

**Table 1:** Characteristics of volunteers

Subject number	Age	Sex (M/F)	Height (cm)	Weight (kg)	${\dot{V}}_{\text{C}{\text{O}}_{2}\text{max}}$ (L/min)	Max workrate (W)	$\Delta P_{\text{E}{\text{T}}_{\text{C}{\text{O}}_{2}}}$ (Torr)
1	27	M	174	77	3.1	285	−3.3
2	25	M	187	84	4.0	315	0.8
3	21	F	173	54	1.6	150	−2.5
4	30	M	192	91	5.0	390	−4.4
5	25	F	158	57	2.0	165	−1.9
6	25	M	179	91	3.6	270	−2.0
7	23	F	173	59	2.7	210	−4.0
8	20	M	182	62	3.5	285	−4.2
9	23	F	170	60	3.0	270	−2.9
10	27	M	179	110	3.6	220	−2.8

Average	25		177	75	3.2	256	−2.7
S.D.	3		9	19	1.0	72	1.5

${\dot{V}}_{\text{C}{\text{O}}_{2}\text{max}}$, maximum oxygen uptake capacity; $\Delta P_{\text{E}{\text{T}}_{\text{C}{\text{O}}_{2}}}$, the effect on $P_{\text{E}{\text{T}}_{\text{C}{\text{O}}_{2}}}$ of 8 h exposure to hypoxia (values are p.m.–a.m.).
